# The effect of teriparatide on patients with atypical femur fractures: a systematic review and meta-analysis

**DOI:** 10.1007/s00402-023-05171-8

**Published:** 2023-12-22

**Authors:** Hazem Mohamed Salamah, Khaled Alsayed Abualkhair, Sara K. Kamal, Hazem A. Mohamed, Ahmad Alkheder, Mohamad Ali Farho, Dillan Mistry, Hany Elbardesy

**Affiliations:** 1 Faculty of Medicine, Zagazig University, Zagazig, 44519 Egypt; 2 Faculty of Medicine, Cairo University, Cairo, Egypt; 3 Department of Otorhinolaryngology, Al Mouwasat University Hospital, Faculty of Medicine, Damascus University, Damascus, Syria; 4 Faculty of Medicine, Syrian Private University, Damascus, Syria; 5 Faculty of Medicine, University of Aleppo, Aleppo, Syria; 6 Department of Orthopaedics Mid Yorkshire Hospitals, Leeds, UK; 7 Department of Trauma and Orthopaedics, Liverpool University Hospitals, Liverpool, UK

**Keywords:** Atypical femoral fracture, Teriparatide, AFF, TPTD, Bone union, Fracture healing

## Abstract

**Introduction:**

Bisphosphonates (BPs) are one of the most often used drugs to lower fracture risk in osteoporosis patients; nonetheless, BPs have been linked to atypical femoral fracture (AFF). Teriparatide (TPTD) is a parathyroid hormone analogue and anabolic drug that may accelerate fracture repair. TPTD has been considered as a possible treatment for AFF, particularly those caused by BP use. We evaluate the effect of TPTD on AFF in this systematic review and meta-analysis.

**Materials and methods:**

A thorough search of: Web of Science, Scopus, PubMed, and Cochrane was conducted on August 2, 2023. Trials evaluating the effect of TPTD on the incidence of: complete bone healing, non-union, early and delayed bone union, progression of incomplete AFF to complete AFF, and time to bone union were included. Using Review Manager (RevMan) version 5.4, the risk ratio (RR) and mean difference (MD) with the corresponding 95% confidence interval (CI) were estimated for dichotomous and continuous outcomes, respectively. The Newcastle–Ottawa Scale was used to assess the quality of studies.

**Results:**

Eight studies met the eligibility criteria and were included in our analysis. TPTD significantly increased the incidence of early bone union (RR = 1.45, 95% CI [1.13, 1.87], *P* = 0.004) and time to bone union (MD = −1.56, 95% CI [−2.86, −0.26], *P* = 0.02) compared to the control group. No significant differences were observed in terms of complete bone healing (RR = 1.09, 95% CI [0.99, 1.13], *P* = 0.12), non-union (RR = 0.48, 95% CI [0.22, 1.04], *P* = 0.06), and progression of incomplete AFF to complete AFF (RR = 0.27, 95% CI [0.04, 1.97], *P* = 0.19).

**Conclusions:**

TPTD is an effective therapy for enhancing and hastening healing following AFF, particularly in postoperative settings. Future large randomized clinical trials are needed to confirm or dispute the results.

**Supplementary Information:**

The online version contains supplementary material available at 10.1007/s00402-023-05171-8.

## Introduction

Atypical femur fractures (AFFs) are a rare complication of anti-resorptive bisphosphonates; such as alendronate or zoledronic acid (BPs). They are used to treat osteoporosis and decrease hip and vertebral fractures. Long-term BP treatment has been linked to decreased bone turnover and remodeling, impairing healing capacity, and predisposing to AFF [1, 2]. The American Society for Bone and Mineral Research (ASBMR) [3] defines AFF; as a fracture that is located distal to the lesser trochanter and just proximal to the supracondylar flare and meets four of the five major criteria: (1) The fracture is associated with minimal or no trauma; (2) the fracture line originates from the lateral cortex and extends transversely or obliquely medially; (3) complete fractures involve both cortices with a medial spike or incomplete fractures involve only the lateral cortex; and (4) the fracture is non-comminuted or minimally comminuted; (5) The fracture site has localized periosteal or endosteal thickening of the lateral cortex (“beaking” or “flaring”).

AFF is frequently resistant to therapy, resulting in poor bone union and a high rate of implant failure [4, 5]. Therefore, AFF is considered a serious health issue with a difficult management and a financial burden on the patients. Patient concern about AFF complications has reduced bisphosphonate use by roughly half in the last decade [6, 7]. The benefits of bisphosphonate therapy in lowering fracture risk, however, outweigh the risk of the AFF. Bisphosphonate therapy reduces bone loss and fracture risk in osteoporosis patients by up to 50% [8]. Therefore, a more effective treatment for such a devastating complication should be sought.

The standard treatment for complete AFF, or intractable pain, is surgery with intramedullary nailing in addition to medical management which includes BPs cessation and assessing dietary calcium and vitamin D status and prescribing adequate supplementation [3]. For incomplete AFF with mild to moderate pain, a trial of conservative therapy with limited weight-bearing could be trialed first [3, 9]. However, surgical treatment is associated with delayed healing and a high rate of revision surgery, whereas conservative treatment typically yields poor results [10, 11].

Teriparatide (TPTD) is an anabolic agent and parathyroid hormone analogue that promotes fracture healing. It is the only FDA-approved anabolic bone in the United States that has been shown to stimulate bone formation and remodeling, thereby accelerating typical fracture healing [12, 13]. TPTD may be a promising treatment for promoting healing of atypical femoral fractures, either alone or in combination with surgical fixation or conservative therapy.

TPTD has been evaluated in several reports; however, the population in most of the available evidence is small, making it difficult to draw firm conclusions about the efficacy of TPTD treatment of AFF patients. In this paper, we conduct a systematic review (SR) and meta-analysis (MA) to determine whether (TPTD) has a significant impact on the incidence of bone union and time to bone union in cases with AFF and to aid in the development of clear guidelines for its use and management of AFF.

## Methods

The authors followed the PRISMA standards for reporting systematic reviews and meta-analyses of randomized controlled trials (RCTs) [14]. This systematic review was registered in the International Prospective Register of Systematic Reviews (PROSPERO), registration identifier CRD42023460067.

### Eligibility criteria

This SR and MA included studies based on the PICOS criteria: patients, intervention, control, outcomes, and study design. The patients of interest were patients with AFFs. The control group consisted of patients who received everything in the intervention group except for TPTD. The studies must report the results of the outcomes of interest to be included. Given the scarcity of controlled studies on the effect of TPTD on AFFs, we searched for studies whose designs were randomized controlled trials as well as comparative observational, prospective, and retrospective studies. There were no restrictions on race, country, publication date, or follow-up duration. To increase our sample size, we included groups with complete or incomplete AFFs, unilateral or bilateral AFFs, and regardless of the site of the femur fracture, such as subtrochanteric or diaphyseal. We included only studies that assessed TPTD after the occurrence of AFFs and were either treated with surgery or conservatively, such as with BP cessation, dietary calcium, and vitamin D.

We excluded single-arm studies, animal studies, conference abstracts, non-English papers, and studies that did not report our outcomes of interest separately for the TPID group.

### Information sources

Relevant articles were identified through a comprehensive search of the PubMed, Web of Science, Cochrane, and Scopus databases from inception to August 2, 2023. Other relevant studies were found by searching the reference lists of the eligible papers.

### Search strategy

A search was conducted in PubMed, Web of Science, Scopus, and Cochrane for comparative studies published using a combination of the following terms: “teriparatide”, “atypical”, “femur”, and “fracture”. There were no filters applied. Supplementary Table 1 contains the complete search strategy for each database.

### Selection process

All records were pooled using Endnote. The data were exported to an Excel sheet, which was subsequently submitted in two stages to find the eligible studies. The title and abstract screening step was carried out first, and records that passed this stage were moved to the full-text screening stage. It is worth noting that the eligibility of each article in each phase was independently examined by two authors. Any disagreements were settled by a third senior author.

### Data collection process

The lead author prepared formatted Excel sheets in which the review authors extracted baseline data and study characteristics, as well as quality assessment and outcomes of interest. Two authors extracted data from each study independently and then discussed it. Any disagreements were settled by a third senior author. Any incomplete or incompatible data were handled using the Cochrane Handbook’s suggested methods [15]

### Data items (outcomes)

The primary outcomes were the incidence of complete bone healing and the time to bone union. Complete bone healing was defined as bridging across three or four cortices and/or disappearance of a visible fracture line on standard antero-posterior and lateral femoral radiographs, and/or a clinical lack of pain at the fracture site on palpation and weight-bearing [16, 17]. It included the incidence of bone healing as reported at the end of the study, regardless of how long it took for the healing to occur. Secondary outcomes included the incidence of early bone union, the incidence of delayed union, the incidence of progression to a complete fracture, and the incidence of non-union. Early bone union was defined if the fracture healed within 6 months. Delayed union was defined as the lack of evidence of bone union within 6 months. Non-union was defined as a fracture that did not achieve union at the end of the study.

### Data items (other variables)

Two authors independently extracted study characteristics and baseline data. Study characteristics included: study ID, study design, follow-up duration, AFF diagnostic criteria applied by each study, laterality, degree, and site of the AFF, AFF treatment employed by the studies, such as surgery or conservative treatment, and description of the intervention group and control group. Baseline data included sample size, age, gender, duration of BP use, number of patients who used BP, number of patients who stopped BP after AFFs, and BP agent used.

### Quality assessment

Two authors independently assessed the quality of the included studies using Newcastle–Ottawa Scale [18]. The quality of the studies was determined by the overall score they received, which was as follows: very good (9–10 points), good (7–8 points), satisfactory (5–6 points), and unsatisfactory (0–4 points).

### Effect measures and synthesis methods

Review Manager (RevMan) version 5.4 [28] was used to conduct all the analyses. All the data was collected as means ± standard deviation (SD), or event and total for continuous and dichotomous outcomes, respectively. The continuous outcome data of time to union was measured using the inverse variance statistic method and reported as mean differences with a 95% confidence interval (CI), and the Mantel–Haensze equation to calculate the pooled RR and 95% CI was used for the remaining dichotomous outcomes. Cochrane’s *Q* test and the *I*^2^ statistic were used to assess heterogeneity. Significant heterogeneity was considered if the *P* value was less than 0.1 and the *I*^2^ was greater than 60%. We used the random effects model regardless of heterogeneity due to differences in studies and patient characteristics, as well as limited data, which did not justify assuming the presence of a true effect size among the included studies and using the fixed effects model. To solve and identify the source of heterogeneity, the leave-one-out strategy was employed. Subgroup analyses were carried out to determine the impact of TPTD on surgically treated cases and cases with complete fractures.

## Results

### Study selection

**Fig. 1:**
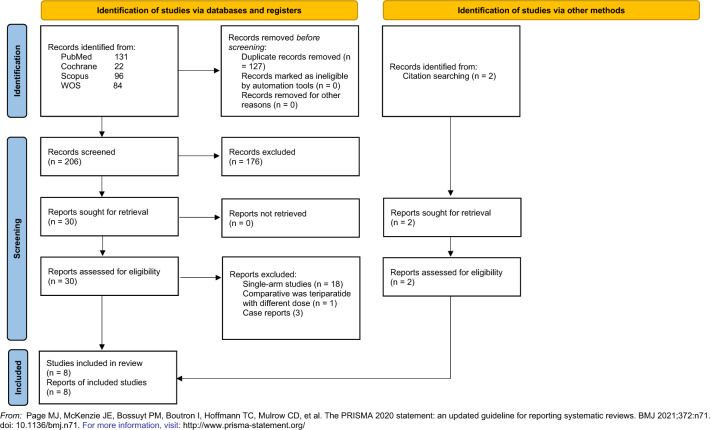
A flowchart shows the detailed process of the search strategy and study selection. From: Page et al. [25]

The database search yielded a total of 333 records. After duplicates were removed, 206 records were entered into the selection process and evaluated for eligibility criteria. Finally, our study included eight eligible studies [16, 17, 19–24]. Figure 1 shows the detailed process of search strategy results and study selection.

### Study characteristics

The meta-analysis included six retrospective studies [17, 19–24] and one prospective study [16], representing a total of 238 patients, 86 of whom received teriparatide and 152 of whom did not. The majority of the included studies were conducted on Asian populations, with the exception of Shin et al. [21] and Chiang et al. [16], which were conducted in the USA and Australia, respectively. One study [22] included patients with incomplete fractures; three studies [17, 19, 24] included patients with complete AFFs; and one study [21] included patients with complete AFFs; however, six patients suffered from contralateral incomplete fractures. In two studies [17, 23], the site of the fracture was diaphyseal, while the majority of the rest of the studies included both diaphyseal and subtrochanteric fractures. Except for Cho et al. [17], which addressed only unilateral fractures, all other studies included both unilateral and bilateral fractures. All patients were treated surgically in five studies [17, 19–21, 24], conservatively in one [22], and surgically or conservatively in the remaining two [16, 23], one of which reported the results separately for patients treated conservatively or surgically [23]. The summary of the study characteristics are shown in Table 1. Almost all of the patients were old females who had been taking BPs prior to the onset of AFFs but had stopped taking them after the onset of AFFs. The most commonly used BP drug was alendronate. The patients characteristics are summarised in Table 2.

**Table 1:** Study characteristics

Study ID	Country (centre)	Study Design	Diagnosis	Definitions	AFF diagnostic criteria	Laterality, degree, and site of the AFF (N of patients for each)	Follow up duration (months)	Treatment of AFFs	Intervention group	Control group
Shin et al. [21]	USA (Two centers)	A retrospective comparative study from January 2010 to October 2017	58 AFFs in 52 patients with complete AFFs	Complete bone healing was defined as full painless weight bearing with bridging callus across at least three cortices on anteroposterior (AP) and lateral views of the femur Non-union was defined as a definite fracture gap after a minimum of 9 months with no visible progressive signs of healing for 3 months	AFF was diagnosed based on radiographic evidence of transverse or short oblique fracture lines, medial spikes, focal lateral cortical thickening, and a relative lack of comminution at the fracture site	Bilateral AFF (6) unilateral AFF (46) complete fracture (52) Incomplete fracture (6) Subtrochanteric fracture (20) Diaphyseal fractures (38)	14	All patients were treated with surgery (closed intramedullary nailing)	Teriparatide, 20 μg, once daily, SC + Calcium and vitamin D supplements for 6.9 ± 2.0 months	Calcium and vitamin D supplements
Miyakoshi et al. [23]	Japan (single-center)	A retrospective comparative study from 2006 to 2013	45 AFFs in 34 patients with AFFs associated related to BPs use for osteoporosis	Complete bone healing was defined as bridging across three or four cortices and/or loss of a visible fracture line based on standard antero-posterior and lateral femoral radiographs Early bone union was defined if the fracture healed within 6 months Delayed union was defined if the fracture healed after more than 6 months but within 2 years Non-union was defined as a fracture that did not achieve union or showed pseudo-joint as of the final follow-up after more than 2 years	According to second report (2014) of the ASBMR task force	Bilateral AFF (11) unilateral AFF (23) complete fracture (32) Incomplete fracture (13) Diaphyseal fractures (45)	Teriparatide = 24.9 ± 23.7 Non-teriparatide = 15.2 ± 3.6	16 AFFs in teriparatide and 21 AFFs in the control group were treated with surgery (intramedullary nail fixation or a locking plate) 5 AFFs in teriparatide and 3 AFFs in the control group were treated conservatively	Teriparatide, 20 μg, once daily in 17 fractures and Teriparatide, 56.5 μg, once weakly in 4 fractures + vitamin D supplements	No teriparatide was given. vitamin D supplements
Yeh et al. [24]	Taiwan (single-center)	A retrospective comparative study from January 2008 and December 2014	16 AFFs in 13 patients with complete AFFs related to BPs use	Delayed union was defined as the lack of evidence of bone union at 6 months postoperatively Non-union was defined as a fractured bone that has not completely healed within 9 months of injury and that has not shown progression of healing on serial radiographs for 3 consecutive months	AFF was diagnosed based on radiographic evidence of transverse or short oblique fracture lines, medial spikes, focal lateral cortical thickening, and a relative lack of comminution at the fracture site	Bilateral AFF (3) unilateral AFF (10) Complete fracture (16) subtrochanteric fracture (10) Proximal femur fracture (6)	12	All patients were treated with surgery (internal fixation with an intramedullary device)	Teriparatide, 20 μg, once daily, SC + Calcium for at least 6 months	Calcium
Chiang et al. [16]	Australia (single-center)	A prospective comparative study from 2009 to 2011	22 AFFs in 14 patients with AFFs related to BPs use	Complete bone healing was considered to have occurred when the fracture line was no longer visible and the patient was pain-free Non-union was defined as persistent fracture lines and pain	Complete atypical femoral fractures were defined as transverse or short oblique fractures without comminution. Incomplete fractures were defined as an incomplete fracture line on the lateral cortex, or abnormalities on technetium bone scan/ MRI suggestive of stress fractures in the lateral cortical region	Bilateral AFF (8) unilateral AFF (6) complete fracture (6) Incomplete fracture (8)	12	5 AFFs in the teriparatide and 6 AFFs in the control were treated conservatively 3 AFFs in the control were treated with surgery	Teriparatide, 20 μg, once daily, SC + Calcium and cholecalciferol for 6 months	Calcium
Png et al. [22]	Singapore (single-center)	A retrospective comparative study from 2002 to 2017	78 AFFs in 69 patients with Incomplete AFFs	Complete bone healing was defined by the disappearance of the visible fracture line	According to second report (2014) of the ASBMR task force	Bilateral AFF (9) unilateral AFF (60) Incomplete fracture (78) Subtrochanteric fracture (30) Diaphyseal fractures (48)	Median (IQR) = 39.7 (19.3–55.0)	All patients were treated conservatively	Teriparatide for at least 1 year	No teriparatide was given
Lee et al. [19]	Korea (Multicenter)	A retrospective comparative study from 2009 to 2014	46 AFFs in 44 patients with complete AFFs related to BPs use	Complete bone healing was defined as the callus bridging of three of the four cortices on anteroposterior and lateral radiographs of the femur Delayed union was defined as radiographic evidence of union that has not been observed until 6 months after surgery Non-union was defined as when complete bony union was not achieved up to 1 year after surgery or implant failure, such as nail breakage, developed during the follow-up period	According to second report (2014) of the ASBMR task force	Bilateral (2) Unilateral (42) Complete fracture (46) Subtrochanteric fracture (15) Diaphyseal fractures (31)	20.1 (range = 6–65)	All patients were treated with surgery (22 AFFs were treated with cephalomedullary nail and 24 AFFs were treated with standard interlocking nail)	Teriparatide	No teriparatide was given
Takakubo et al. [20]	Japan (Multicenter)	A retrospective comparative study from 2009 to 2014	11 AFFs in 8 patients with AFFs related to BPs use	NA	According to second report (2014) of the ASBMR task force	Bilateral AFF (3) unilateral AFF (5) Subtrochanteric fracture (7) Diaphyseal fractures (4)	28 (range = 12–70)	All patients were treated with surgery (10 AFFs with intramedullary nail fixation and one AFFs using a locking plate)	Teriparatide with low-intensity pulsed ultrasonography	Low-intensity pulsed ultrasonography without teriparatide
Cho et al. [17]	Korea (single-center)	A retrospective comparative study from 2007 to 2015	16 AFFs in 16 patients with complete diaphyseal AFFs	Complete bone healing was defined as clinical absence of pain at the fracture site on both palpation and weight-bearing and radiological evidence of bridging of 3 or more cortices on 2 different views	According to second report (2014) of the ASBMR task force	Unilateral AFF (16) Complete fracture (16) Diaphyseal fractures (16)	33.9 ± 26.5	All patients were treated with surgery (internal fixation using plates)	Teriparatide for at least 3 months + Calcium and vitamin D complex	Calcium and vitamin D complex

**Table 2:** Patients baseline characteristics

Study ID	Groups	Sample size	Age	Female	Duration of BPs use (months)	No. of Patients used BPs	No. of patients stopped BPs after AFFs	BPs drug (N. of patients used it)
			Mean ± SD	*N* (%)	Mean ± SD	*N* (%)	*N* (%)	
Shin et al. [21]	Teriparatide	28	74.8 ± 9.8	28 (100)	38.4 ± 27.6	18 (64.3)	28 (100)	Alendronate (11), Risedronate (4), Ibandronate (3), Zoledronic acid (4)
	Non teriparatide	30	73.8 ± 7.4	30 (100)	33.6 ± 24	22 (73.3)	30 (100)	Alendronate (9), Risedronate (4), Ibandronate (3), Zoledronic acid (2)
Miyakoshi et al. [23]	Teriparatide	21	79.9 ± 3.3	21 (100)	60.0 ± 29.1	21 (100)	21 (100)	Alendronate (17), Risedronate (4)
	Non teriparatide	24	77.0 ± 5.9	24 (100)	44.7 ± 34.8	16 (100)	16 (100)	Alendronate (15), Risedronate (9)
Yeh et al. [24]	Teriparatide	8	70.25 ± 68	8 (100)	52.32	8 (100)	8 (100)	Alendronate (8)
	Non teriparatide	8	69.25 ± 72.5	8 (100)	48	8 (100)	8 (100)	Alendronate (8)
Chiang et al. [16]	Teriparatide	5	77.5 ± 1.6	13 (92.8)	96	5 (100)	5 (100)	Alendronate (11), Risedronate (1), sequential Pamidronate/Zoledronate (2)
	Non teriparatide	9	77.3 ± 1.3		72	9 (100)	9 (100)	
Png et al. [22]	Teriparatide	4	68.5 ± 10.4	68 (98.6)	60.3 ± 31.8	65 (98.5)	43 (54)	NA
	Non teriparatide	72						
Lee et al. [19]	Teriparatide	14	70.1 ± 6.75	44 (100)	61.2 ± 42	46 (100)	14 (100)	NA
	Non teriparatide	32					21 (65.6)	
Takakubo et al. [20]	Teriparatide	5	54.9 ± 20.13	11 (100)	52 ± 33.8	11 (100)	4 (80)	Alendronate (5)
	Non teriparatide	3					2 (66.7)	Alendronate (3), Risedronate (2), Minodronate (1)
Cho et al. [17]	Teriparatide	6	75.9 ± 6.9	16 (100)	47.1 ± 30.1	8 (50)	8 (100)	NA
	Non teriparatide	10						NA

### Quality assessment

All of the studies included were of good quality (7–8 points). Seven studies had a total of eight points [16, 17, 19–21, 23, 24], while only one study received seven points [22]. The quality assessment of the studies included is shown in Supplementary Table 2.

## Results of syntheses

### Incidence of complete healing

**Fig. 2:**
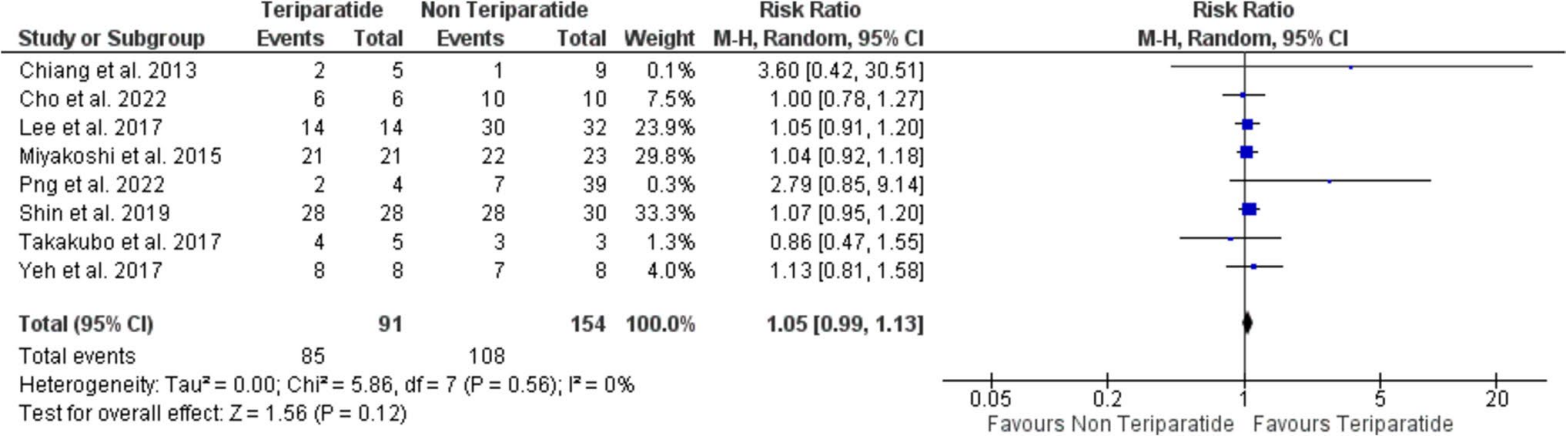
A forest plot shows the risk ratio of complete healing

The analysis included eight studies [16, 17, 19–24], with a total of 91 patients in the teriparatide arm and 154 patients in the non-teriparatide arm. The analysis showed insignificant increase in the incidence of complete bone healing in patients who received teriparatide compared to those who didn’t (RR = 1.09, 95% CI [0.99, 1.13], *P* = 0.12). The pooled analysis was homogenous (*P* = 0.56, *I*^2^ = 0%) (Fig. 2).

### Time to bone union (months)

**Fig. 3:**
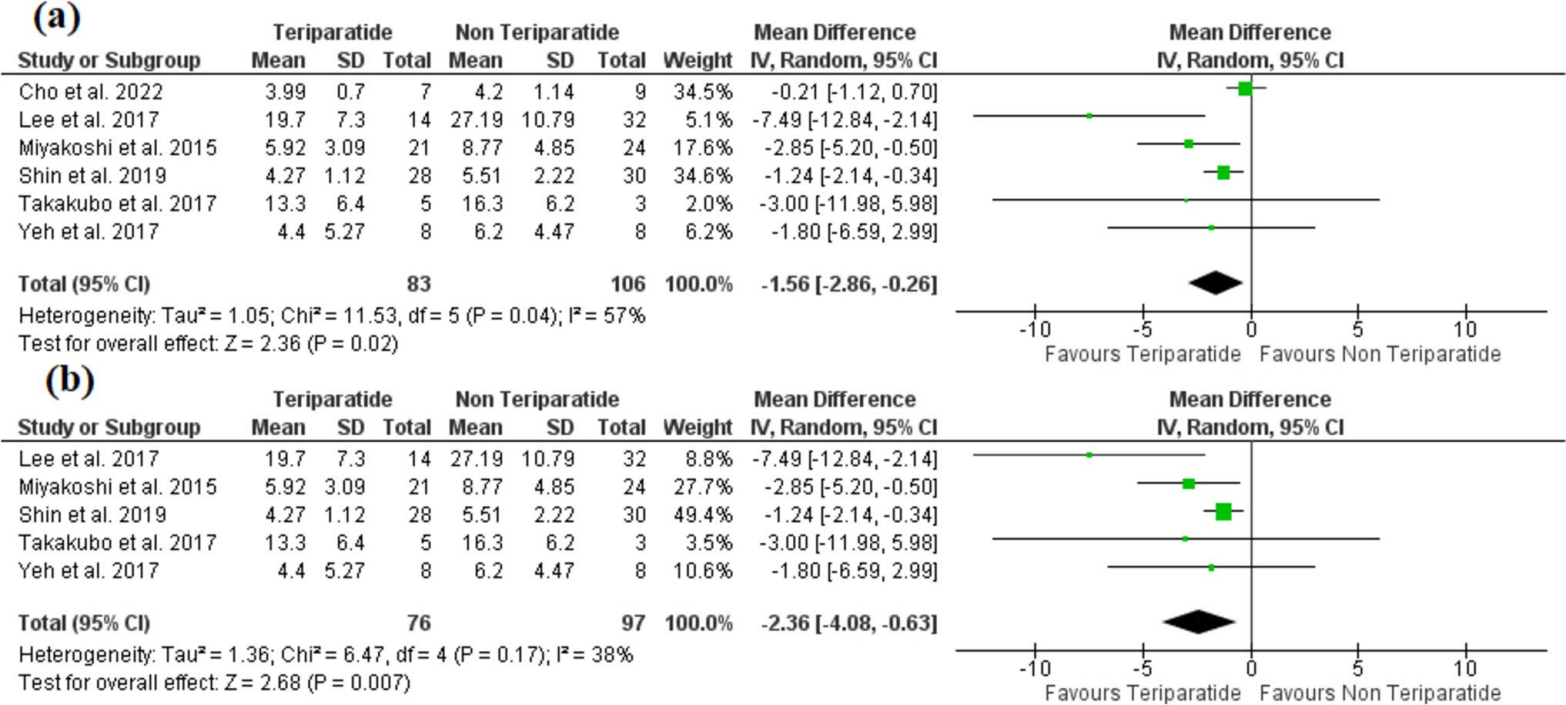
A forest plot shows the mean difference in the time to bone union (**a**). **b** The results after exclusion Cho et al. [17] study

The analysis included six studies [17, 19–21, 23, 24], with a total of 83 patients in the teriparatide arm and 106 patients in the non-teriparatide arm. The time required for bone union to occur was significantly reduced by TPTD (MD = −1.56, 95% CI [−2.86, −0.26], *P* = 0.02) (Fig. 3a). The pooled analysis was heterogeneous (*P* = 0.04, *I*^2^ = 57%), which was resolved after the exclusion of Cho et al. [17] (*P* = 0.17, *I*^2^ = 38%) without significant effect on the overall estimate (MD = −2.36, 95% CI [−4.08, −0.63], *P* = 0.007) (Fig. 3b).

### Incidence of non-union

**Fig. 4:**
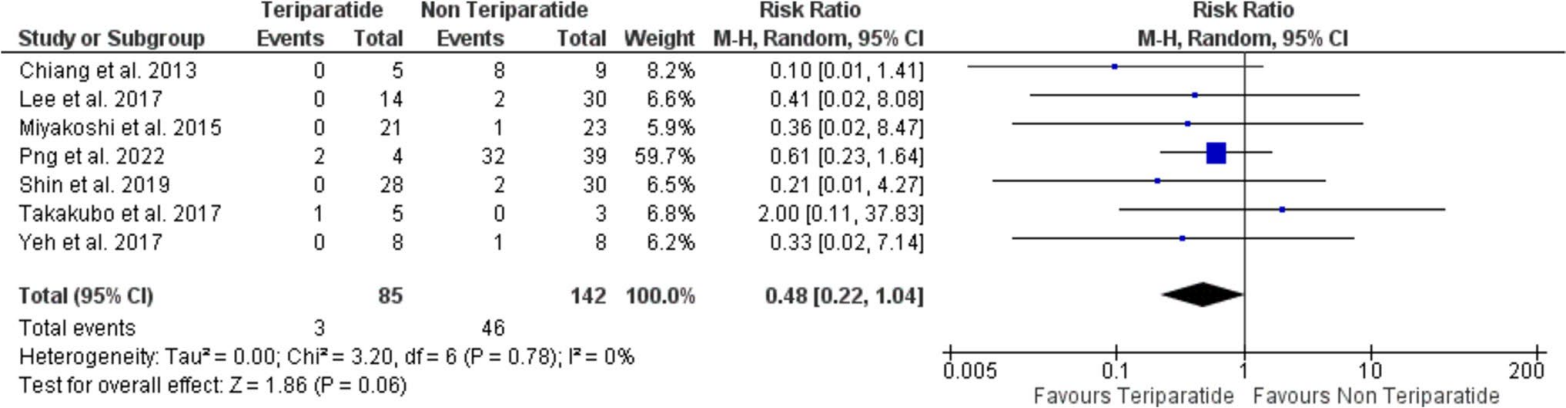
A forest plot shows the risk ratio of non-union

The analysis included seven studies [16, 19–24], with a total of 85 patients in the teriparatide arm and 142 patients in the non-teriparatide arm. The analysis showed that TPTD insignificantly reduced the incidence of bone non-union (RR = 0.48, 95% CI [0.22, 1.04], *P* = 0.06). The pooled analysis was homogenous (*P* = 0.78, *I*^2^ = 0%) (Fig. 4).

### Incidence of early and delayed union

**Fig. 5:**
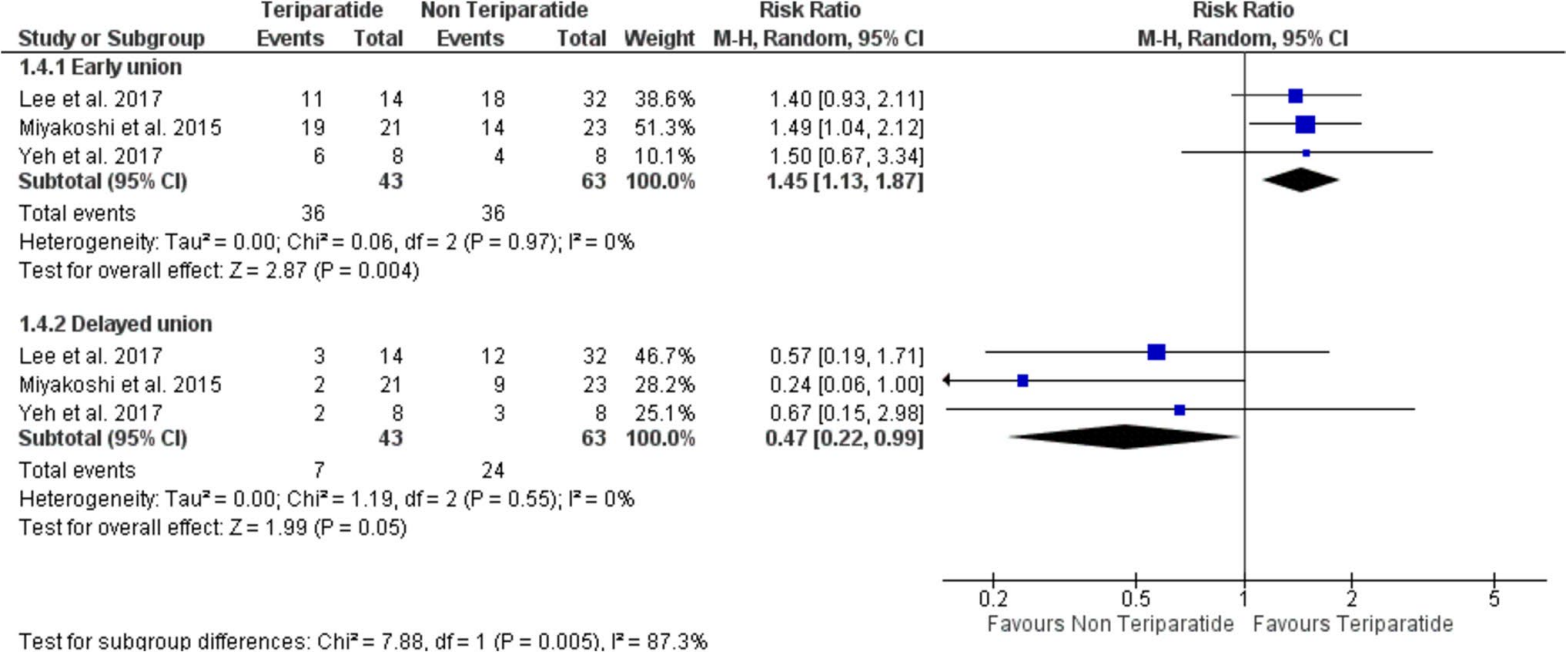
A forest plot shows the risk ratio of early bone union and delayed bone union

The analysis included three studies [19, 23, 24] with a total of 43 patients in the teriparatide arm and 63 patients in the non-teriparatide arm. The analysis found that the incidence of bone healing within 6 months of TPTD treatment in patients who received TPTD was significantly higher than that in the control group (RR = 1.45, 95% CI [1.13, 1.87], *P* = 0.004). The pooled analysis was homogenous (*P* = 0.97, *I*^2^ = 0%) (Fig. 5). The incidence of delayed bone healing, on the other hand, was significantly lower in the TPTD group compared to the control group (RR = 0.47, 95% CI [0.22, 0.99], *P* = 0.05). The pooled analysis was homogenous (*P* = 0.55, *I*^2^ = 0%) (Fig. 5).

### Incidence of progression to complete fracture

**Fig. 6:**
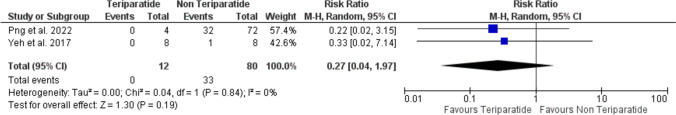
A forest plot shows the risk ratio of progression to complete fracture

The analysis included three studies [22–24] with a total of 12 patients in the teriparatide arm and 80 patients in the non-teriparatide arm. TPTD lowered the incidence of progression to complete fracture but insignificantly (RR = 0.27, 95% CI [0.04, 1.97], *P* = 0.19). The pooled analysis was homogenous (*P* = 0.84, *I*^2^ = 0%) (Fig. 6).

### Subgroup analysis

Subgroup analysis showed that in patients who received surgical treatment, TPTD demonstrated a significantly shorter time to bone union (MD = −1.72, 95% CI [−3.09, −0.35], *P* = 0.01). Furthermore, it significantly increased the incidence of early bone union in patients undergoing surgical treatment and those with complete fractures (RR = 1.52 95% CI [1.16, 1.98], *P* = 0.002) and (RR = 1.54 95% CI [1.18, 2.01], *P* = 0.001). Table 3 shows the results of subgroup analysis.

**Table 3:** Subgroup analyses

Outcomes	Subgroups	No of studies	Pooled RR (95% CI)	*P* value (overall effect)	*I*^2^ (%)	*P* value (heterogeneity)
Incidence of complete healing	Surgically treated	6 (77/104)	1.05 (0.98, 1.13)	0.16	0	0.97
	Complete AFF surgically treated	4 (39/71)	1.04 (0.95, 1.14)	0.40	0	0.97
Time to union	Surgically treated	**6 (78/103)**	**−1.72 (−3.09, −0.35)**	**0.01**	**62**	**0.02**
	Complete AFF surgically treated	4 (40/70)	−2.70 (−5.58, 0.18)	0.07	78	0.007
Non-union	Surgically treated	5 (71/92)	0.49 (0.13, 1.88)	0.30	0	0.87
	Complete AFF surgically treated	3 (40/70)	0.44 (0.07, 2.54)	0.36	0	0.96
Early union	Surgically treated	**3 (38/61)**	**1.52 (1.16, 1.98)**	**0.002**	**0**	**0.86**
	Complete AFF surgically treated	**3 (33/61)**	**1.54 (1.18, 2.01)**	**0.001**	**0**	**0.80**
Delayed union	Surgically treated	3 (38/61)	0.48 (0.22, 1.09)	0.08	0	0.47
	Complete AFF surgically treated	3 (33/61)	0.51 (0.22, 1.19)	0.12	0	0.45

Bold values indicate statistical significance at the *P* < 0.05 level

## Discussion

Osteoporosis is considered the most common bone disease [26]. BP is one of the most widely used medications to reduce fracture risk in osteoporosis patients. Although BP usage has been linked to the development of AFFs, ASBMR estimates the absolute risk of BP-associated AFFs to be low. Ranging from 3.2 to 50 cases per 100,000 person-years [3]. AFFs, if occurred, can be extremely burdensome for the patient and have a negative impact on their social and economic status. Therefore, a treatment should be proposed to cope with AFF situations related to the critical use of BPs. We conducted a systematic review and meta-analysis to look into TPTD as a potential therapy to alleviate this burden.

Teriparatide is a PTH analogue that binds to PTH type 1 receptors, increasing osteoblast survival and quantity, resulting in trabecular and cortical bone formation. This mechanism of action contrasts sharply with that of antiresorptive drugs, such as bisphosphonates, which reduce osteoclast-mediated bone resorption while also inhibiting new bone development because resorption and formation are inextricably linked processes [27]. Therefore, TPTD has been proposed as a potential therapy for BP-associated AFFs.

A previous literature review conducted by Gao et al., which investigated case reports and observational studies, concluded that TPTD enhances AFF healing by shortening the time to bone union and decreasing the incidence of non-union [28]. However, due to the limited data in their study, they didn’t perform a MA. Here, we included more controlled studies and performed the first MA to provide more robust, reliable data. The analysis indicated that TPTD can significantly reduce the time required for bone union while also increasing the likelihood of early bone union within 6 months. However, contrary to the conclusions of Gao et al., the effect of TPTD on reducing the incidence of non-union and increasing the likelihood of complete healing was not statistically significant.

In 2005, a study was conducted to measure the mean time needed for a typical femoral fracture to heal, which was approximately 3 months [29]. AFFs require a longer period of time. Egol et al. [29] conducted a retrospective study of 41 complete and displaced atypical bisphosphonate-associated femoral fractures treated surgically with intramedullary nails. The mean time of bone healing was 8.3 months. Our analysis suggests that using TPTD, this period could be reduced to less than 6 months.

Peich et al. 2011 employed TPTD to treat pubic bone fractures in elderly osteoporotic patients. According to their findings, the average time to bone union in the TPTD group was roughly 8 weeks, compared to nearly 13 weeks in the control group [30]. Nonetheless, Aspenberg et al. used TPTD to treat distal radius fractures in postmenopausal women in order to reduce the time required for bone union [12]. Therefore, our findings may not be exclusive to femoral fractures.

Gomberg et al. [29] used TPTD along with vitamin D and calcium to treat a 63-year-old postmenopausal female patient in an attempt to accelerate the healing process. And after one year, the patient no longer needed narcotics for her pain. Gomberg et al. [29] stated that the healing process could have been spontaneous and related to time. This is further supported by the fact that we didn’t find significant differences between TPTD and the control in terms of the incidence of complete healing at the end of the follow-up period. Therefore, TPTD may be considered if the healing process needs to be sped up. It should be noted, however, that the incidence of complete bone healing and nonunion in our study favored the teriparatide group, though this did not reach statistical significance, and larger trials are needed to confirm the findings.

Based on our subgroup analysis, teriparatide effectively increased the incidence of early bone healing and decreased the time to bone union in AFF after surgical repair, as well as in those with complete AFF. However, due to a lack of data, the outcomes of conservatively treated patients could not be evaluated. Miyakoshi et al. [23] performed a subgroup analysis based on treatment modality and discovered significant differences in healing time in surgically treated patients receiving TPTD versus the control group, but not in non-surgically treated patients receiving conservative therapy. However, the sample size was too small to draw solid conclusions. Therefore, our findings support the use of TPTD to accelerate bone healing postoperatively, regardless of the extent of the fracture. Future large trials are needed to assess the effect of TPTD on patients receiving conservative therapy instead of surgery.

The analysis revealed that TPTD insignificantly reduced the incidence of the progression of incomplete AFF to complete AFF. This could be attributed to the small sample size included in the analysis. However, it could refer to the inability of TPTD to heal conservatively treated incomplete AFFs and that it is only effective in postoperative settings. Further research is needed to address this question.

The findings have important implications for future guidelines and clinical decision-makers. TPTD can be considered in situations where the healing process of AFFs needs to be sped up. The patients can be given the option that healing may occur spontaneously, but TPTD will shorten the time needed for bone union to occur.

### Strengths and limitations

This SR and MA benefit from a thorough search that includes the most recent relevant trials as well as all accessible data, which was either examined as primary, secondary, or exploratory outcomes. Furthermore, all of the pooled analyses were homogeneous, and we performed sensitivity analysis to test the results’ robustness in the presence of heterogeneity. However, our meta-analysis is not without limitations. The small sample size is the main concern. Furthermore, there were variations in the characteristics of the included studies and patients. However, we employed a random effect model to provide more robust results and performed a subgroup account for some of these variations. However, due to limited data, we couldn’t perform further subgroup analyses.

## Conclusion

TPTD significantly shortened the time to bone union and increased the incidence of early bone healing compared to the control group. However, the effect on the incidence of overall complete healing or non-union is minimal. We present TPTD as a postoperative treatment to hasten and enhance the healing process. Further studies with a large sample size are required to validate or refute these findings.

### Supplementary Information

Below is the link to the electronic supplementary material.

Supplementary file1 (DOCX 20 KB)

## Data Availability

All data analyzed during this study are included in this published article or listed in references.
