# Supplementary material for: The effect of teriparatide on patients with atypical femur fractures: a systematic review and meta-analysis
Source: Arch Orthop Trauma Surg. 2023 Dec 22;144(3):1091–106. doi: 10.1007/s00402-023-05171-8 (PMC10896930; doi:10.1007/s00402-023-05171-8)
Supplement: Supplementary file 1 — Supplementary file1 (DOCX 20 KB) [file 402_2023_5171_MOESM1_ESM.docx]

**The effect of teriparatide on patients with atypical femur fractures: A systematic review and meta-analysis.**

Hazem Mohamed Salamah^1*^, Khaled Alsayed Abualkhair^1^, Sara K. Kamal^2^, Hazem A. Mohamed^1^, Ahmad Alkheder^3,4^, Mohamad Ali Farho^5^, Dillan Mistry ^6^, Hany Elbardesy^7^

1 Faculty of Medicine, Zagazig University, Zagazig, Egypt

2 Faculty of Medicine, Cairo University, Cairo, Egypt

3 Department of Otorhinolaryngology, Al Mouwasat University Hospital, Damascus, Syria

4 Faculty of Medicine, Syrian Private University, Damascus, Syria.

5 Faculty of Medicine, University of Aleppo, Aleppo, Syria

6 Department of orthopaedics Mid Yorkshire hospitals, Leeds, UK

7 Department of Trauma and Orthopaedics, Liverpool University Hospitals, Liverpool, UK

***Corresponding author:**

Hazem Mohamed Salamah. Affiliation: Faculty of Medicine, Zagazig University, Zagazig, 44519, Egypt.

Email: [hazem.salamah@gmail.com](mailto:hazem.salamah@gmail.com)

**Supplementary table 1.** Full search strategy for each database

| Database | Search Terms | Search Field | Search Results |
| --- | --- | --- | --- |
| PubMed | (Teriparatide OR hPTH OR Human Parathyroid Hormone OR Parathar OR Teriparatide Acetate OR Forteo) AND (atypical) AND (femur fractures OR femur fracture) | All Fields | 140 |
| Cochrane | (Teriparatide OR hPTH OR Human Parathyroid Hormone OR Parathar OR Teriparatide Acetate OR Forteo) AND (atypical) AND (femur fractures OR femur fracture) | All Fields | 22 |
| WOS | (Teriparatide OR hPTH OR Human Parathyroid Hormone OR Parathar OR Teriparatide Acetate OR Forteo) AND (atypical) AND (femur fractures OR femur fracture) | All Fields | 93 |
| SCOPUS | TITLE-ABS-KEY ((teriparatide OR hpth OR "Human Parathyroid Hormone" OR parathar OR "Teriparatide Acetate" OR forteo) AND (atypical) AND ("femur fractures" OR "femur fracture")) | Title, Abstract, Keywords | 106 |

| Study | Selection | | | | Comparability | Exposure | | | Total |
| --- | --- | --- | --- | --- | --- | --- | --- | --- | --- |
|  | Representativeness of the Exposed Cohort | Selection of the Non-Exposed Cohort | Ascertainment of Exposure | Demonstration That Outcome of Interest Was Not Present at Start of Study | Comparability of Cohorts | Assessment of Outcome | Was Follow-Up Long Enough for Outcomes to Occur | Adequacy of Follow Up of Cohorts | Total number of stars |
| Shin et al. 2019 |  | * | * | * | ** | * | * | * | 8 |
| Miyakoshi et al. 2015 |  | * | * | * | ** | * | * | * | 8 |
| Yeh et al. 2017 |  | * | * | * | ** | * | * | * | 8 |
| Chiang et al. 2013 |  | * | * | * | ** | * | * | * | 8 |
| Png et al. 2022 |  | * | * | * | * | * | * | * | 7 |
| Lee et al. 2017 | * | * | * | * | * | * | * | * | 8 |
| Takakubo et al. 2017 | * | * | * | * | * | * | * | * | 8 |
| Cho et al. 2022 |  | * | * | * | ** | * | * | * | 8 |

**Supplementary table 2.** Newcastle-Ottawa Scale (NOS) for assessing the quality of included studies
